# Comparison of Common Methods for Precision Volume Measurement of Hematoma

**DOI:** 10.1155/2020/6930836

**Published:** 2020-07-17

**Authors:** Minhong Chen, Zhong Li, Jianping Ding, Xingqi Lu, Yinan Cheng, Jiayun Lin

**Affiliations:** ^1^College of Science, Zhejiang Sci-Tech University, Hangzhou, Zhejiang, China 310018; ^2^The Affiliated Hospital of Hangzhou Normal University, Hangzhou, Zhejiang, China 310015; ^3^College of Science, Southern University of Science and Technology, Shenzhen, Guangdong, China 518055

## Abstract

**Purpose:**

Our aim is to conduct analysis and comparison of some methods commonly used to measure the volume of hematoma, for example, slice method, voxelization method, and 3D-Slicer software method (projection method).

**Method:**

In order to validate the accuracy of the slice method, voxelization method, and 3D-Slicer method, these three methods were first applied to measure two known volumetric models, respectively. Then, a total of 198 patients diagnosed with spontaneous intracerebral hemorrhage (ICH) were recruited. The patients were split into 3 different groups based on the hematoma size: group 1: volume < 10 ml (*n* = 89), group 2: volume between 10 and 20 ml (*n* = 59), and group 3: volume > 20 ml (*n* = 50). And the shape of the hematoma was classed into regular (round to ellipsoid) with smooth margins (*n* = 76), irregular with frayed margins (*n* = 85), and multilobular (*n* = 37). The slice method, voxelization method, and 3D-Slicer method were adopted to measure the volume of hematoma, respectively, considering the nonclosed models and the models which may contain inaccurate normal information during CT scan. Moreover, the results were compared with the 3D-Slicer method for closed models.

**Results:**

There was a significant estimation error (*P* < 0.05) using these three methods to calculate the volume of the closed hematoma model. The estimated hematoma volume was calculated to be 14.2086743 ± 0.900559087 ml, 14.2119130 ± 0.900851812 ml, and 14.2123825 ± 0.900835916 ml using slice method 1, slice method 2, and the voxelization method, respectively, compared to 14.212656 ± 0.900992371 ml using the 3D-Slicer method. The mean estimation error was *-*0.00398172 ml, *-*0.00074303 ml, and *-*0.00027354 ml caused by slice method 1, slice method 2, and voxelization method, respectively. There was a significant estimation error (*P* < 0.05), applying these three methods to calculate the volume of the nonclosed hematoma model. The estimated hematoma volume was calculated to be 14.1928246 ± 0.902210314 ml using the 3D-Slicer method. The mean estimation error was calculated to be *-*0.00402121 ml, *-*0.00078237 ml, -0.00031288 ml, and *-*0.01983136 ml using slice method 1, slice method 2, voxelization method, and 3D-Slicer method, respectively.

**Conclusions:**

The 3D-Slicer software method is considered as a stable and capable method of high precision for the calculation of a closed hematoma model with correct normal direction, while it would be inappropriate for the nonclosed model nor the model with incorrect normal direction. The slice method and voxelization method can be the supplement and improvement of the 3D-Slicer software method, for the purpose of achieving precision medicine.

## 1. Introduction

Intracerebral hemorrhage (ICH) has been identified as a significant cause of death and disability around the world [1]. The increasing incidence of cerebral hemorrhage can cause progression of the disease. In addition, the amount of cerebral hemorrhage, or the cerebral hematoma volume, can be taken as a major indicator of early mortality at the time of admission. It is also among the most effective indicators of the degree of neurological recovery within 90 days of the onset of the disease [2–6].

**Figure 1:**
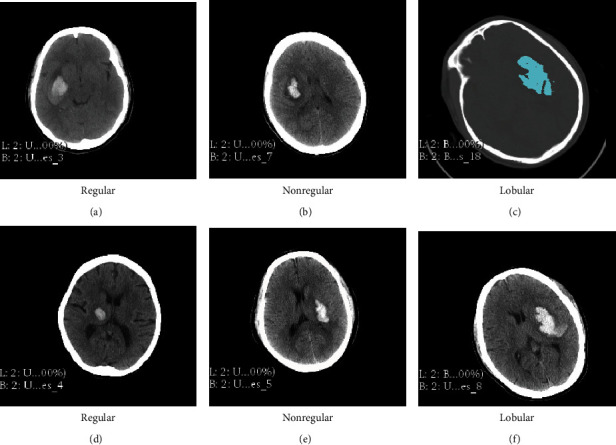
Hematoma shape classification.

The diversity of hematoma shapes is one of the primary causes of errors in applying volume assessment methods. In practice, there will be brain lesions with inconspicuous lesions, irregular borders, discontinuities, and high noise. The shape of hypertensive cerebral hemorrhage can be categorized into kidney shape, round shape, oval shape, fusiform shape, and irregular shape, as shown in Figure 1. The diversity of hematoma shapes (Figure 1) makes it necessary to apply volumetric calculation methods that ensure both accuracy and robustness. Therefore, in order to facilitate the accurate diagnosis and treatment of disease, choosing an accurate, simple, and noninvasive approach to the measurement of intracranial hematoma volume is definitely conducive to the selection of treatment options, evaluation of clinical outcomes, and prediction of disease progression.

There are various methods to measure the volume of hematoma, and they are mainly classed into four categories, including the mathematical formula method, tool measurement method, CT machine measurement method, and software method. Among them, the Tada formula method is one of most commonly used formula methods. The formula is *V* = 1/2 × *A* × *B* × *C*, where *A* indicates the long diameter, *B* represents the broad diameter, and *C* denotes the number of hematoma layers. The Tada formula has been extensively applied to assess the volume of intracerebral hematoma. Since the Tada formula in theory is derived from the ellipsoid volume formula, when the shape of an intracranial hematoma shows similarity to an ellipsoid, which has a regular shape, a hematoma such as a “ball” shape can be calculated using this method. However, when the shape of an intracranial hematoma is distant from the ellipsoid, that is, irregular hematoma or lobular hematoma, the Tada formula performs poorly [7]. In order to address this drawback, some improved ball volume formulas [8] were proposed based on the Tada formula. In spite of this, the accuracy of calculation for the volume formula remains associated with the shape of the hematoma. The more irregular the hematoma morphology, the more significant the error in the calculation results.

As computer technology progresses at a fast pace, the hematoma model can be measured and analyzed using different software methods. The 3D-Slicer method is one of the software methods purposed to measure the volume of a hematoma. It provides a free open source software platform for biomedical research to be conducted (http://www.slicer.org). With regard to the measurement principle, it is similar to the computer-aided volume analysis. The software is capable of identifying hematoma pixels based on CT data in cerebral hemorrhage images and reconstructing blood clots in a three-dimensional manner. Besides, it is free from restriction by hematoma morphology and bleeding sites. The 3D-Slicer method could ensure both accuracy and simplicity for hematoma assessment [9], which makes it gradually accepted as an effective measurement method [10–12]. In addition, the 3D-Slicer software method has been demonstrated to be faster and less user-intensive compared to manual delineation, which makes it suitable as a standard method. Xu et al. [7] analyzed the accuracy of the Tada formula by comparing with the 3D-Slicer software method, which led to the conclusion that hematoma assessment with software 3D-Slicer is a low-cost, accurate, and effective technique for the measurement of ICH volume. However, the stability of the 3D-Slicer software method has not yet been included in discussion. As for measurement of ICH volume, some other methods can be analyzed and applied as well, such as the slice method and voxelization method.

In this paper, our aim is to improve the accuracy of hematoma assessment. The stability of the 3D-Slicer method was analyzed, and a comparison was performed between the 3D-Slicer method and two other methods. It was found out that, when the three-dimensional hematoma model is nonclosed or the surface normal of the hematoma model is incorrect, the 3D-Slicer method will give rise to some errors, which can be rectified by two other methods, the slice method and the voxelization method.

## 2. Commonly Used Methods

### 2.1. 3D-Slicer Method (Projection Method)

Jointly developed by Harvard University Brigham and Women's Hospital and the Massachusetts Institute of Technology, 3D-Slicer software represents a free open source software platform for biomedical research. Hematoma is reconstructed using the original DICOM format data in 3D-Slicer software according to CT scanning, which ensures an accurate measurement for hematoma. Besides, the triangular mesh model is used for the volume measurement of hematoma by the 3D-Slicer method, slice method, and voxelization method.

#### 2.1.1. Operation

**Figure 2:**
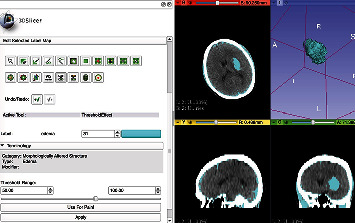
The hematoma model was reconstructed using 3D-Slicer software.

Run 3D-Slicer software (3D-Slicer 4.6.2, Harvard University, USA), import the CT data of the patient in DICOM format, adjust the size of image, and proceed as follows: run Editor → Threshold → Apply. The CT threshold range is manually set, while the software automatically identifies and marks the pixels that constitute the hematoma. If necessary, editing is continued to completely separate the hematoma from the surrounding normal brain tissue. Run MakeModel → Models. Then, the three-dimensional shape of the hematoma and the volume of the hematoma can be determined, as shown in Figure 2.

#### 2.1.2. Principle

3D-Slicer software, as developed for the processing of image visualization and image analysis, is premised on VTK, ITK, Teem, QT, and other open source software [9, 13]. The principle of volume measurement is similar to the computer-aided volume analysis. The hematoma is segmented using the GrowCut method [9]. The hematoma volume is calculated following the three-dimensional reconstruction of hematoma. This method is simple, accurate, and resistant to the impact made by the shape and location of hematoma [14].

Its volume calculation is performed by referencing the volume calculation formula in the open source software VTK, where the major class for the calculation of volume and area in VTK is vtkMassProperties [15]. The principle of this method is premised on the triangulation projection, which means that the model volume refers to the algebraic sum of the convex polyhedral volume enclosed by all triangular patches and the projection plane.

It is assumed that the coordinates of each triangle vertex are *P*_0_(*x*_0_, *y*_0_, *z*_0_), *P*_1_(*x*_1_, *y*_1_, *z*_1_), *P*_2_(*x*_2_, *y*_2_, *z*_2_), the length of the triangle edge are *a*, *b*, and *c*, the normal of the triangular patch is *u* (*u*_*x*_, *u*_*y*_, *u*_*z*_), and the center of gravity of the triangular patch is avg(*x*, *y*, *z*). Then, the projection volume is expressed as

(1) $$\begin{array}{c} V_{x}=\text{area}\cdot u_{x}\cdot \text{av}{\text{g}}_{x},\\ V_{y}=\text{area}\cdot u_{y}\cdot \text{av}{\text{g}}_{z},\\ V_{z}=\text{area}\cdot u_{z}\cdot \text{av}{\text{g}}_{z}, \end{array}$$

where $\text{area}=\sqrt{\left|s\cdot \left(s-a\right)\cdot \left(s-b\right)\cdot \left(s-c\right)\right|}$ means the triangular area and *s* = (*a* + *b* + *c*)/2. Therefore, the calculation formula for model volume is written as

(2) $$\begin{array}{c} V=\left|k_{x}\cdot V_{x}+k_{y}\cdot V_{y}+k_{z}\cdot V_{z}\right|, \end{array}$$

where *V*_*x*_, *V*_*y*_, and *V*_*z*_ denote the sums of the projection volumes for the triangular patches, while *k*_*x*_, *k*_*y*_, and *k*_*z*_ represent the weights of each projection direction.

The measurement by 3D-Slicer software provides an accurate and simple method for the hematoma volume based on CT data. As shown in experiment, intracranial hematoma clearance (only about 2.71 ml left in average) is performed in combination with 3D-Slicer software, which achieves a 93.8% clearance rate [16]. However, it is discovered that the hematoma model required for the 3D-Slicer software method must be the closed triangular mesh model, and accurate normal information of the model surface needs to be known in advance. In some cases, the hematoma model may be nonclosed or with incorrect normal information before the volume measurement. For example, when the boundary of a tumor surrounds that of the hematoma data, there is a possibility that the hematoma model is not closed. When the Marching Cube algorithm is applied to reconstruct the three-dimensional hematoma model, it will also give rise to the situation where the surface normal is inaccurate. Therefore, measuring the volume with the 3D-Slicer method in these cases will result in a significant error.

### 2.2. Slice Method

This method firstly performs layering on the three-dimensional hematoma model, then calculates the area of the corresponding section, and estimates the model volume based on the distance between adjacent planes. The idea of slicing is to measure the volume of hematoma by the sum of quantitative measurement between consecutive sections; that is, the hematoma volume calculation formula is obtained as *V* = ∑*S*_*i*_ × *h*, where *S*_*i*_ indicates the area of each CT slice and *h* denotes the thickness of the CT slice. The volume is determined based on the accumulation, which means that the three-dimensionally reconstructed hematoma is sliced, the adjacent section is supposed to form a round table, and the volume of all the sliced round tables is added as the total volume of the model. Different formulas can be obtained to calculate the volume of hematoma by applying different methods to calculate the volume of the round table *V*_*i*_. For example, see the following.

*Slice method 1*: formula for each sliced round table volume is expressed as

(3) $$\begin{array}{c} V_{i}=\left(S_{i1}+S_{i2}+\sqrt{S_{i1}S_{i2}}\right)\frac{\text{step}}{3}, \end{array}$$

where *S*_*i*1_ represents the upper floor area, *S*_*i*2_ indicates the lower floor area, and step refers to the interval between two slices.

*Slice method 2*: formula for each sliced round table volume is shown as follows:

(4) $$\begin{array}{c} V_{i}=\left(S_{i1}+S_{i2}\right)\frac{\text{step}}{2}, \end{array}$$

where *S*_*i*1_ indicates the upper floor area, *S*_*i*2_ refers to the lower floor area, and step denotes the interval between two slices.

From the aforementioned volume calculation formulas, it can be known that the calculation of the hematoma volume is related to the slice thickness (slice interval). A small thickness can improve accuracy, but this incurs more computation costs. Conversely, a large thickness reduces computation costs, but this causes accuracy to be compromised. Therefore, how to identify the appropriate slice thickness (interval) is a major problem facing the use of the slice method.

### 2.3. Voxelization Method

Voxelization provides a modeling method that approximates the geometric shape of a three-dimensional model by using spatial voxel units. These spatial voxels show similarity to pixels in a two-dimensional image and can be regarded as the expansion from a two-dimensional square area to a three-dimensional cube unit.

The realization of the voxelization method for the volume measurement involves two aspects. The octree operation is firstly implemented, and then, the calculation of boundary voxel volume is optimized. The major details are as follows:

1. *Implementation of the Octree Operation*. (a) The bounding box of the models is computed. (b) The octree is subdivided, the voxel with no intersection with the model mesh as a leaf voxel is marked, and the nonleaf voxel is subdivided again. (c) All leaf voxels are determined as either inside or outside the model. (d) The volume is defined as the sum of the volume of all inside leaf voxels and boundary voxels (i.e., the lowest nonleaf voxels).
2. *Optimization of the Boundary Voxel Volume*. The volume of the boundary voxel (the lowest nonleaf voxel) can be calculated using the slice method.

According to the voxelization method, spatial voxel units are required to approximate the three-dimensional model, and the computational complexity is higher compared to the 3D-Slicer software method (projection method) and the slice method. The computational accuracy of the voxelization method is determined by the size of the voxel unit and the volume calculation of the boundary voxel. When the model volume is unknown, the results obtained by the voxelization method can be taken as the reference to compare the accuracy between the slice method and the 3D-Slicer software method.

### 2.4. Comparison of Three Measurement Methods

#### 2.4.1. Standard Models with Known Volume

Firstly, a comparison is performed between the 3D-Slicer method, the slice method, and the voxelization method for the volume measurement of standard models, the volumes of which are known. For the slice method and voxelization method, we firstly calculate the volume with a large interval and a large voxel unit, respectively, and then reduce the interval and the voxel unit by a certain value until the calculated volume is relatively stable.

*(1) Volume Measurement for Quadrangular Pyramid Model.* It is assumed that the length of a pyramid is *l* = 30, the width is *w* = 30, and the height is *h* = 20. Then, the volume of a pyramid is calculated to be 6000 using the quadrangular pyramid volume formula. The model is triangulated to construct a nonclosed 3D model and a closed 3D model with different triangular facets, respectively. Then, the 3D-Slicer method, the slice method, and the voxelization method are applied to measure the quadrilateral pyramid models, respectively. The results are indicated in Table 1.

*(2) Volume Measurement for Cube.* Suppose the length of the cube is *a* = 10, it can be known intuitively that the volume is 1000. Similarly, the model is triangulated to obtain a nonclosed 3D model and a closed 3D model with different triangular facets. Then, the 3D-Slicer method, the slice method, and the voxelization method are employed to measure the volumes, respectively. The results are presented in Table 1 as well.

#### 2.4.2. Nonstandard Models with Unknown Volume

Two 3D models of hematomas stemming from the patients were first reconstructed using 3D-Slicer software. Besides, the 3D-Slicer method, the slice method, and the voxelization method are applied to measure the volumes of the nonclosed 3D model and the closed 3D model, respectively. The results are shown in Table 2.

#### 2.4.3. Discussion

1. As for the closed cube model, the 3D-Slicer method is capable of ensuring accuracy. In comparison with the 3D-Slicer method, the results obtained by the slice method and the voxelization method show their accuracy and minor errors
2. The slice method and the voxelization method are consistent for either closed or nonclosed models. In addition, the results as obtained by the slice method and the voxelization method show similarity to the 3D-Slicer method applied for the closed model. However, the 3D-Slicer software method could result in a significant estimation error for the nonclosed model
3. The voxelization method and the slice method exhibit a low level of sensitivity to the number of triangular facets, and the volumes are identical for the model with different facets. The 3D-Slicer method shows sensitivity to the closeness of the model, and a small reduction of the facets will lead to a large error

## 3. Hematoma Volume Measurement and Comparison Analysis

For all patients, the 3D hematoma models were reconstructed using the 3D-Slicer software. Then, volume measurements were performed using the 3D-Slicer method, the slice method, and the voxelization method, respectively.

### 3.1. Materials and Methods

#### 3.1.1. Patients

In this study, the patients admitted to the Affiliated Hospital of Hangzhou Normal University between December 2017 and January 2018 with diagnosis of spontaneous ICH were recruited. A total of 198 consecutive patients were recruited, including 132 male patients and 66 female patients, with the average age of 56.2 ± 28.8. The patients with multiple sites of ICH were excluded from this study. All cases were included to the standard that the onset to head CT examination time is less than 24 hours.

#### 3.1.2. Imaging

A total of 198 brain computed tomographic image data sets were acquired according to the hospital PACS system with the digital imaging standard in medicine format.

#### 3.1.3. Patient Groups

The patients were split into 3 different groups depending on the hematoma size. Group 1 was comprised of 89 patients with volume < 10 ml, group 2 consisted of 59 patients with volume ranging from 10 to 20 ml, and group 3 was made up of 50 patients with volume > 20 ml. Based on the maximal slice, the shape of the hematoma was classed into regular (round to ellipsoid) with smooth margins (76 cases), irregular with frayed margins (85 cases), and multilobular (37 cases).

#### 3.1.4. Statistical Analysis

All of the statistical analyses were conducted with SPSS Statistics 21 (IBM Corporation, America). Moreover, GraphPad Prism was applied to draw charts. The relationship between the hematoma volume and the measurement method was analyzed by applying the simple linear correlation. Subsequent to the confirmation of distribution, the data were indicated as the mean ± SD, and unpaired *t*-test or 1-way ANOVA was conducted for comparison between different methods and groups, while the LSD method was applied to compare the two groups. A value of *P* < 0.05 was treated as statistically significant.

### 3.2. Results

We set the volumes of the closed models measured by the 3D-Slicer method as the standard values. The slice method (slice methods 1 and 2), voxelization method, and 3D-Slicer software method were compared using the closed hematoma and nonclosed hematoma models. For different methods, a simple correlation analysis was conducted under different models.

**Figure 3:**
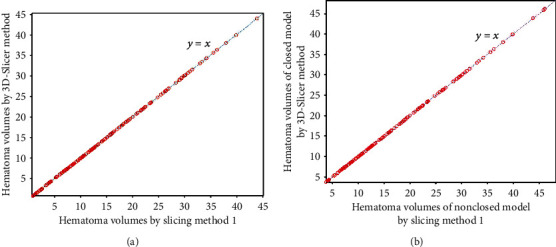
Comparisons of slice method 1 for measuring closed and nonclosed hematoma with the 3D-Slicer method.

**Figure 4:**
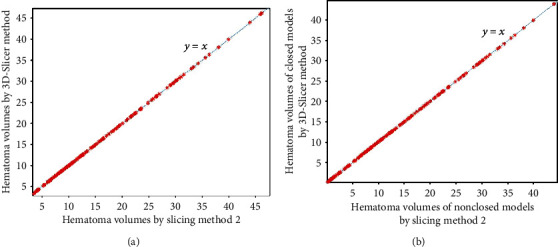
Comparisons of slice method 2 for measuring closed and nonclosed hematoma with the 3D-Slicer method.

**Figure 5:**
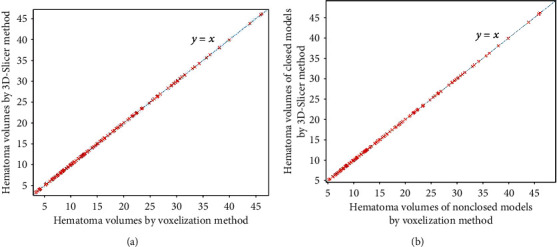
Comparisons of the voxelization method for measuring closed and nonclosed hematoma with the 3D-Slicer method.

**Figure 6:**
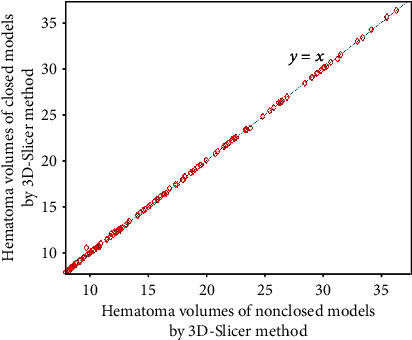
Comparisons of the 3D-Slicer method for measuring closed and nonclosed hematoma.

Figures 3–6 show the comparison results obtained by slice method 1, slice method 2, voxelization method, and 3D-Slicer method for the closed and nonclosed hematoma models. The results displayed in (a) are those for closed hematoma models. The scatter plots shown in Figures 3–6 have demonstrated that the results obtained from slice method 1, slice method 2, and voxelization methods are linearly related to those from the 3D-Slicer method. Moreover, their correlation is close to one. As revealed by the linear correlation analysis carried out by SPSS, the correlation coefficients between the slice methods 1 and 2, the voxelization method, and the 3D-Slicer method for the closed hematoma model were *r* = 1. There were statistically significant differences (*t* = −5.627, *P* < 0.01) observed for the results between the slice method, the voxelization method, and the 3D-Slicer method. From the results in Figures 3–5, we can see that the figures in (b) are similar to the results in the figures in (a). That means the slice methods 1 and 2 and voxelization method are stable when the hematoma model was nonclosed, and the measurement results conform to those of the 3D-Slicer method when the hematoma model is closed. However, large errors will be caused by applying the 3D-Sclicer method to the nonclosed hematoma model.

### 3.3. Analysis

**Figure 7:**
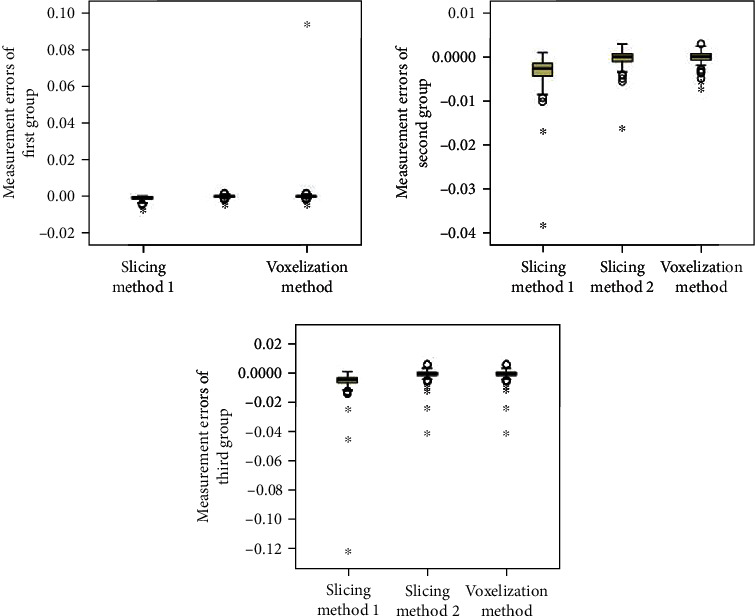
Distribution of measurement errors of closed hematoma models by slice method 1, slice method 2, voxelization method, and 3D-Slicer method (grouping by size).

**Figure 8:**
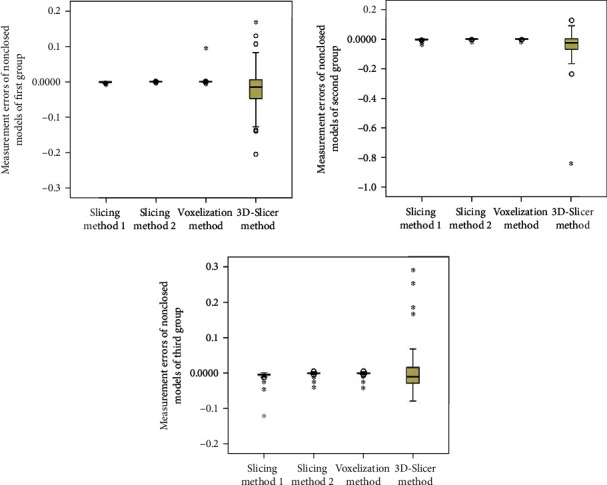
Distribution of measurement errors of nonclosed hematoma models by slice method 1, slice method 2, voxelization method, and 3D-Slicer method (grouping by size).

When the patients are split into groups based on hematoma size, the statistical analyses are shown in Tables 3 and 4 and Figures 7 and 8. We can see that the mean errors of the results obtained by using the slice methods 1 and 2 and the voxelization method for closed and nonclosed hematoma measurements are broadly the same. The mean error of the voxelization method is less significant compared to the mean error of the slice method. The 3D-Slicer software method measures the nonclosed hematoma model with a significantly higher error than the slice method and the voxelization method. Specifically, for the first group, the error caused by the 3D-Slicer measurement for the nonclosed model is 18 times that of the voxelization method. For the second group, the error by the 3D-Slicer measurement for the nonclosed model exceeds 100 times that of the voxelization method.

**Figure 9:**
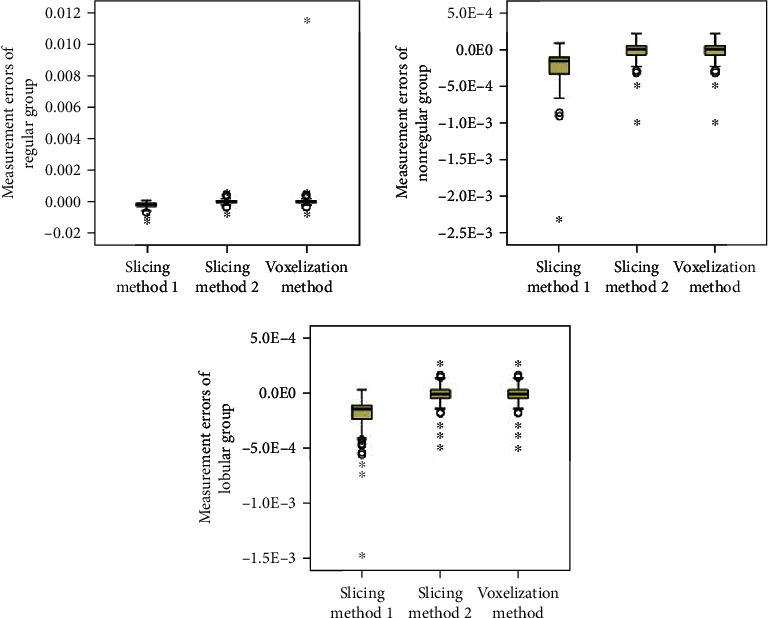
Distribution of measurement errors of closed hematoma models by slice method 1, slice method 2, voxelization method, and 3D-Slicer method (grouping by shape).

**Figure 10:**
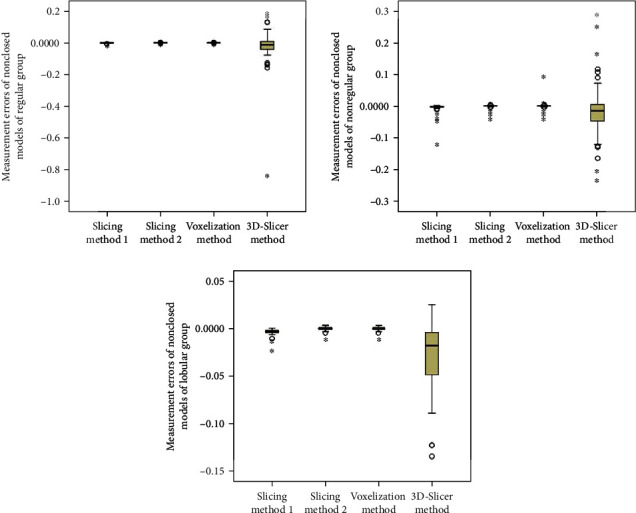
Distribution of measurement errors of nonclosed hematoma models by slice method 1, slice method 2, voxelization method, and 3D-Slicer method (grouping by shape).

When the hematoma is classed by shape, the statistical analyses are shown in Tables 5 and 6 and Figures 9 and 10. It can be found out that slice methods 1 and 2 and voxelization methods are unaffected by the shape of the hematoma, and the measurement results obtained by these methods do not cause significant errors due to the irregular shape of hematoma. The mean errors as measured by slice method 1 and voxelization method show the same order of magnitude for the regular group and the irregular group. Besides, the voxelization method measures the hematoma of the lobulated group with less error, with its order of magnitude reaching 10^−6^. Compared with the voxelization method, the 3D-Slicer method measures the nonclosed hematoma with significant errors. The error of the regular group, irregular group, and lobular group measured by the 3D-Slicer method is shown to be 151 times, 15 times, and nearly 1000 times that of the voxelization method, respectively.

## 4. Discussion

The accurate measurement of hematoma volume is of clinical significance as hematoma volume has been commonly used to correlate with treatment strategy, functional outcome, and mortality. It is inevitable for an inaccurately assessed hematoma volume to exert influence on the initial treatment decisions, thus leading to an undesirable outcome. Meanwhile, hematoma volume plays a crucial role in the prognosis of patients. The measurement of hematoma volume after cerebral hemorrhage can be taken as a potential indicator for prediction, which is of great significance to the clinical development of a sensible treatment. There are various forms of cerebral hemorrhage, especially for the presence of irregular hematoma, which makes it necessary to find an accurate method to determine the size of the volume based on different hematoma morphologies.

At present, the widely used methods to measure the volume of hematoma include the 3D-Slicer method and the Tada formula method. The Tada formula method is considered to be a rough calculation of hematoma due to its inaccuracy in measuring irregular hematoma [7]. Moreover, the 3D-Slicer method is unaffected by the shape and location of the hematoma. Due to its real-time efficiency and low requirements on the operator, the 3D-Slicer method has been widely applied to measure the volume of hematoma.

For precision medicine, the 3D-Slicer method and other popular approaches to the volume measurement of hematoma were studied in this paper. The 3D-Slicer method caused a significant error in the measurement of the nonclosed hematoma model or the model with wrong surface normal information. Nevertheless, the slice method and voxelization method were unaffected by closeness of the hematoma model nor the model with wrong normal information. Therefore, they can be treated as effective supplement methods of the 3D-Slicer method to measure the volume of hematoma. The drawbacks shown by slice and voxelization methods are the slice interval and the division of voxel units which affect both efficiency and accuracy. If there are significant errors between the 3D-Slicer method and the slice method (or the voxelization method), the voxelization method (or slice method) can be applied to validate the accuracy of these methods of measurement.

## Figures and Tables

**Table 1:** Volumes of pyramid and cubic models by voxelization, slice, and 3D-Slicer methods.

Model	Facets	Segments	Voxel unit	Slice method		Voxelization method	3D-Slicer method
				Method 1	Method 2		
Pyramid (closed)	2048	7	0.46875	6000.00	6000.18	6000.18	6000.00
		8	0.23438	6000.00	6000.04	6000.04	

Pyramid (unclosed)	1792	7	0.46875	6000.00	6000.18	6000.18	4625.18
		8	0.23438	6000.00	6000.04	6000.04	

Cubic (closed)	2304	7	0.15625	1000.00	1000.00	1000.00	1000.00
		8	0.07813	1000.00	1000.00	1000.00	

Cubic (unclosed)	2088	7	0.15625	1000.00	1000.00	1000.00	943.12
		8	0.07813	1000.00	1000.00	1000.00	

**Table 2:** Volumes of hematoma models by voxelization, slice, and 3D-Slicer methods.

Model	Facets	Segments	Voxel unit	Slice method		Voxelization method	3D-Slicer method
				Method 1	Method 2		
Hematoma 1 (closed)	13436	7	0.427	6131.83	6135.47	6135.47	6134.42
Hematoma 1 (unclosed)	13433	7	0.427	6131.83	6135.46	6135.46	6085.78
Hematoma 2 (closed)	2424	7	0.212	1056.25	1056.54	1056.54	1056.18
Hematoma 2 (unclosed)	2421	7	0.212	1056.25	1056.54	1056.54	929.30
Hematoma 3 (closed)	12582	7	0.599	12104.49	12107.78	12107.78	112107.68
Hematoma 3 (unclosed)	12579	7	0.599	12104.48	12107.77	12107.77	11872.43

**Table 3:** Mean errors of closed hematoma (grouping by size) by slice and voxelization methods compared with the 3D-Slicer method.

	*n*	Slice method 1	Slice method 2	Voxelization method
First group	89	0.00125517	0.00220450	0.00082404
Second group	59	0.00405220	0.00067305	0.00067305
Third group	50	0.08751800	0.00175580	0.00175580

**Table 4:** Mean errors of nonclosed hematoma (grouping by size) by slice, voxelization methods, and 3D-Slicer method, compared with the 3D-Slicer method for closed models.

	*n*	Slice method 1	Slice method 2	Voxelization method	3D-Slicer method for nonclosed models
First group	89	0.00128517	0.00025000	0.00079449	0.01426753
Second group	59	0.00405797	0.00067915	0.00036763	0.04990034
Third group	50	0.00888480	0.00185180	0.00131520	0.00574640

**Table 5:** Mean errors of closed hematoma (grouping by shape) by slice and voxelization methods compared with the 3D-Slicer method.

	*n*	Slice method 1	Slice method 2	Voxelization method
Regular group	76	0.00188145	0.00019487	0.00019487
Irregular group	85	0.00601259	0.00155753	0.00046388
Lobular group	37	0.00363027	0.00000216	0.00000216

**Table 6:** Mean errors of nonclosed hematoma (grouping by shape) by slice, voxelization methods, and 3D-Slicer method, compared with the 3D-Slicer method for closed models.

	*n*	Slice method 1	Slice method 2	Voxelization method	3D-Slicer method for nonclosed models
Regular group	76	0.00193513	0.00024842	0.00013171	0.01995789
Irregular group	85	0.00605059	0.00159624	0.00109388	0.01590706
Lobular group	37	0.00364405	0.00000946	0.00002892	0.02858676

## Data Availability

The data used to support the findings of this study are included within the article.
